# Unveiling the Role of Oxidative Stress in Cochlear Hair Cell Death: Prospective Phytochemical Therapeutics against Sensorineural Hearing Loss

**DOI:** 10.3390/ijms25084272

**Published:** 2024-04-12

**Authors:** Nicholas B. Gill, Presley D. Dowker-Key, Mark Hedrick, Ahmed Bettaieb

**Affiliations:** 1 Department of Nutrition, University of Tennessee Knoxville, Knoxville, TN 37996-0840, USA; 2 Department of Audiology & Speech Pathology, The University of Tennessee Health Science Center, Knoxville, TN 37996-0240, USA; 3 Graduate School of Genome Science and Technology, University of Tennessee Knoxville, Knoxville, TN 37996-0840, USA; 4 Department of Biochemistry, Cellular and Molecular Biology, University of Tennessee Knoxville, Knoxville, TN 37996-0840, USA

**Keywords:** hearing loss, cochlear hair cells, inflammation, oxidative stress, diabetes, apoptosis, phytochemical

## Abstract

Hearing loss represents a multifaceted and pervasive challenge that deeply impacts various aspects of an individual’s life, spanning psychological, emotional, social, and economic realms. Understanding the molecular underpinnings that orchestrate hearing loss remains paramount in the quest for effective therapeutic strategies. This review aims to expound upon the physiological, biochemical, and molecular aspects of hearing loss, with a specific focus on its correlation with diabetes. Within this context, phytochemicals have surfaced as prospective contenders in the pursuit of potential adjuvant therapies. These compounds exhibit noteworthy antioxidant and anti-inflammatory properties, which hold the potential to counteract the detrimental effects induced by oxidative stress and inflammation—prominent contributors to hearing impairment. Furthermore, this review offers an up-to-date exploration of the diverse molecular pathways modulated by these compounds. However, the dynamic landscape of their efficacy warrants recognition as an ongoing investigative topic, inherently contingent upon specific experimental models. Ultimately, to ascertain the genuine potential of phytochemicals as agents in hearing loss treatment, a comprehensive grasp of the molecular mechanisms at play, coupled with rigorous clinical investigations, stands as an imperative quest.

## 1. Introduction

### 1.1. Hearing Loss

Hearing loss (HL) has far-reaching consequences on a person’s life, impacting psychological, emotional, societal, and economic aspects. The costs of hearing aids and specialized services further contribute to the economic burden faced by individuals with HL. Addressing the impacts of hearing loss requires comprehensive support systems, awareness campaigns, and accessible resources to enrich the quality of life for individuals with hearing impairment. In this review, we explore the physiological, biochemical, and molecular aspects of HL, alongside the current and emerging therapeutic strategies, outlining their potential benefits.

By 2050, approximately 2.5 billion individuals are expected to experience some level of hearing impairment, and at the minimum hearing rehabilitation will be warranted for 700 million individuals. Notably, the prevalence of hearing loss surpasses that of diabetes or cancer, being twice as frequent [1]. The incidence of hearing loss rises with age and is most pronounced among males, individuals of White ethnicity, those with lower income, and individuals with lower educational attainment [1]. The elderly represent the most vulnerable population for hearing impairment; however, unhealthy listening practices put over 1.1 billion young adults at risk of permanent yet preventable hearing loss [2]. At speech frequencies that commonly present in older adults, hearing loss is correlated with emotions of loneliness and social isolation stemming from its impact on communication and social interactions [3,4]. This isolation can lead to negative psychiatric consequences, such as depression, distress, and anxiety, which are often reported alongside more severe hearing loss [5,6]. Moreover, there is a well-established relationship between age-related hearing loss (ARHL) and cognitive decline, with ongoing research exploring the causal mechanism. Among modifiable risk factors for dementia, including diabetes mellitus, hypertension, and smoking, hearing loss has the highest relative risk for the development of dementia throughout a person’s life [7]. Two hypotheses have been investigated to understand the connection between hearing loss and cognitive decline: one suggests that cognitive decline precedes hearing loss, and the other attributes hearing loss as the precursor to cognitive decline [8]. In addition to the implications for psychiatric disorders and cognitive decline, a study investigating findings reported by the National Health and Nutrition Examination Survey conducted from 1999 to 2007 revealed that speech-frequency hearing loss is significantly associated with increased heart disease mortality and overall death rates [9]. Hearing loss should not be viewed solely as an impairment of the sensory system but as a condition with wide-ranging consequences for an individual’s overall health. Unfortunately, as the global population ages in the coming decades, hearing loss and its related health issues pose a growing concern.

### 1.2. Statistics and Classifications of Hearing Loss

In the U.S., self-reported incidences of hearing difficulties can reach as high as 16.8% of the adult population, equivalent to 43.4 million adults [10]. Although self-reporting may inflate the frequency of hearing impairment, low rates of hearing screening and referrals to audiology specialists might underestimate the prevalence in the U.S. According to one study, only 14.6% of primary care physicians regularly perform hearing screenings, potentially leading to missed diagnoses [10]. Moreover, out of the 48.8 million individuals who sought medical attention for hearing complaints in a 5-year period, only 60% were referred to otolaryngologists or audiologists for continued treatment. According to the American Academy of Otolaryngology–Head and Neck Surgery, the shortage of otolaryngologists and audiologists, especially in rural areas, is a significant concern. The current distribution of medical professionals tends to favor urban areas, leaving rural patients at a disadvantage in accessing hearing specialists. This scarcity may hinder access to specialized care, and it does not meet forecasted demand as the US population ages, thus leading to delayed treatment and worsening of hearing impairment [11]. Consequently, many patients remain undiagnosed and untreated despite the increasing prevalence of hearing loss, primarily due to the low rates of screening, referrals, and limited access to specialized care.

In low-income countries, the accessibility to hearing services is severely limited, with 78% and 93% having fewer than one otolaryngologist and audiologist per million people, respectively [12]. Without a large initiative to improve healthcare infrastructure in these regions, this disparity will continue to worsen, as the World Health Organization (WHO) estimates that over the next three decades the greatest increase in hearing loss will be seen in the African and eastern Mediterranean regions [13]. To best address the frequency and extent of hearing loss across the globe, the Global Burden of Disease Expert Group in Hearing Loss recommends an improved classification system. This new system defines seven distinct grades of bilateral hearing impairment (Table 1), each with functional descriptions, and includes a category for unilateral hearing impairment [14]. However, without a substantial initiative to enhance healthcare infrastructure in these regions, this disparity is likely to persist.

The World Report on Hearing observed that individuals with moderate hearing impairment (≥35 dB threshold shift in the better ear) face the highest risk of adverse effects and stand to benefit significantly from medical interventions like hearing aids and cochlear implants if their hearing loss progresses further [13,15]. Moderate hearing loss can be considered the threshold for disabling hearing loss, which affects approximately 27% (430 million) of individuals with hearing impairments [12]. Since age is the most significant risk factor for hearing loss, regions with an aging population are projected to have greater increases in individuals experiencing disabling hearing loss [13]. An increase in disabling hearing loss along with the disparity in hearing specialists and severely low rates of hearing aid use do not confer hopeful predictions about the future state of hearing health.

Unfortunately, the usage of hearing aids in the U.S. is dismally low (28.8 million), with only 15% of individuals who meet the criteria for hearing aids actually using them [15,16]. Notably, the U.S. rate of hearing aid usage is even lower than the global estimates provided by the World Report on Hearing, which indicate that only 17–23% of eligible individuals worldwide use hearing aids [2]. The reasons for such low usage are not solely related to healthcare access but also involve factors like cost, usability challenges, and the unfortunate social stigma associated with wearing hearing aids [15]. To combat the increasing number of people with hearing impairments, there is an urgent need for improved management that addresses disparities in access to hearing specialists, the utilization of hearing aids and cochlear implants, and the availability of therapies to protect hearing. Advancements in prevention and treatment of hearing loss require a comprehensive understanding of different types of hearing loss and the underlying mechanisms of auditory dysfunction.

### 1.3. Types of Hearing Loss

Hearing loss is categorized into three groups, based on which part of the auditory system is affected. These categories are known as conductive, sensorineural, and mixed hearing loss. When considering years lost to disability, hearing loss ranks as the third most significant contributor, and among sensory impairments, it causes the highest number of years lost [13].

#### 1.3.1. Conductive Hearing Loss (CHL)

CHL occurs when the outer and middle ear fail to convert sound waves into mechanical vibrations that are transmitted to the inner ear [17]. The process of conducting sound involves sound waves entering the auditory canal, causing the tympanic membrane to vibrate, which, in turn, transmits the vibrations through the ossicular chain to the oval window and into the cochlea. Damage or blockage in any of these structures can result in CHL. For example, the auditory canal may be blocked by an accumulation of cerumen, exostosis, or Otitis externa [15]. The tympanic membrane can develop perforations due to acoustic trauma, leading to sclerosis, which reduces the membrane’s flexibility and sensitivity to sound waves [15]. Additionally, the ossicular chain can be disrupted by conditions such as cholesteatoma, Otitis media with effusions, and otosclerosis, thereby interrupting the conduction of sound waves from the auditory canal to the cochlea [15]. Given that the inner ear remains generally unaffected in CHL cases, once this conductive barrier is surpassed, individuals often experience clear hearing.

#### 1.3.2. Sensorineural Hearing Loss (SNHL)

SNHL occurs when the inner ear is unable to convert fluid waves into auditory nerve signals due to damage in either the cochlear epithelium or auditory nerve and may range from a mild loss to profound hearing loss. The causes of SNHL are varied, with congenital factors including cochlear malformations, auditory nerve deficiencies, and abnormalities in the internal auditory canal [18]. Acquired causes comprise Meniere’s disease, age, noise exposure, and ototoxic medications [15,19,20].

ARHL, commonly known as presbycusis, represents the most prevalent form of SNHL. It typically develops gradually and can be worsened by factors such as exposure to loud noise, usage of ototoxic medications, and other ear-related disorders [21,22]. In presbycusis, higher frequency sounds are affected first with progression to the mid and lower frequencies, leading to a decline in the overall quality of life [5]. In addition to worsening outcomes in ARHL, noise- and ototoxic medication-induced hearing loss are discussed as primary causes of SNHL. Noise-induced hearing loss (NIHL) presents as bilateral loss of hearing and is of chief concern in occupational settings [21,23]. Ototoxicity from medications is not limited to but largely investigated with the antineoplastic cisplatin and aminoglycoside antibiotics [24].

On the other hand, sudden SNHL is a distinct condition characterized by an acute loss of at least 30 dB in hearing within three separate instances over a period of 72 h [15]. Unlike presbycusis, this sudden form of SNHL can be treated according to current clinical guidelines, which recommend a combination of steroid therapy and hyperbaric oxygen therapy [25]. In pediatric patients, progressive SNHL can be associated with genetic conditions or chronic infections, while fluctuating symptoms can also occur due to Meniere’s disease or endolymphatic hydrops [21].

#### 1.3.3. Mixed Hearing Loss

Mixed hearing loss manifests with aspects of both CHL and SNHL, either separately or interconnected. Various conditions can affect both the middle and inner ears, such as otosclerosis, chronic infections, and trauma to the temporal bone [21]. However, for the rest of this review, the primary focus will be on SNHL, as it is the most prevalent type of hearing loss.

## 2. Cellular Mechanisms of Cochlear Hair Cell Degeneration

Over the years, the cellular mechanisms of SNHL have been slowly elucidated to identify major pathways contributing to the pathology. A pervasive commonality among the three major categories is the loss of cochlear hair cells and spiral ganglion neurons; however, the deterioration of these cell groups is attributed to separate and overlapping mechanisms.

Traditionally, it was believed that outer hair cells (OHCs) are the most vulnerable to insults. However, emerging research suggests that in ARHL models, cochlear synaptopathy and spiral ganglion cell degeneration may occur before actual hair cell loss [26]. Noise exposure, even without causing permanent threshold shifts (i.e., noticeable hearing loss) and hair cell death, may exacerbate this process [26,27,28]. Neuronal losses alone are associated with decreased sound discrimination in noisy environments, a condition termed “hidden hearing loss” [29,30]. Over time, accumulated neuronal losses due to aging and exposure to environmental noise can lead to threshold shifts, but it is estimated that this occurs only after losing 80–90% of connections with inner hair cells (IHCs) [30]. Another possible mechanism involves noise-induced glutamate excitotoxicity, which may lead to progressive spiral ganglion cell degeneration by disrupting essential neurotrophin signaling from hair cells [29].

While the concept of hidden hearing loss is intriguing, a full conformation in humans remains challenging due to difficulties in measuring synaptic degeneration [31]. Currently, supporting data for this hypothesis, apart from animal models, are limited to archival temporal bone studies [32,33]. The mechanisms behind cochlear hair cell degeneration are also not completely understood but provide a foundation for comprehending the pathogenesis of SNHL. Studies suggest that early permanent threshold shifts are associated with dysfunction and cell death of OHCs, most commonly in the basal cochlea [34,35]. Recent investigations have revealed that both basal and apical OHCs are lost in the cochleae of aged humans and rats [33,36]. However, even though OHCs deteriorate in both tonotopic regions, the loss of high-frequency hearing precedes the progression into lower frequencies. This phenomenon might be linked to the OHCs’ capability to amplify high-frequency sounds by 50–70 dB, compared to only 20 dB in low frequencies. As a result, threshold shifts would be most significant in the higher frequencies [37].

To protect itself from permanent threshold shifts caused by noise exposure, the cochlea induces temporary threshold shifts, akin to physiological habituation. After noise exposure, temporary threshold shifts occur through the activation of the purinergic receptor P2X 2 ion channel [38]. This channel opens, leading to a reduction in the endocochlear potential around the hair cells, which, in turn, decreases signal transduction and lowers noise sensitivity [39,40]. Additionally, in guinea pigs, noise-induced temporary threshold shifts have been shown to upregulate Bcl-xL, an anti-apoptotic Bcl-2 family member [41].

**Figure 1:**
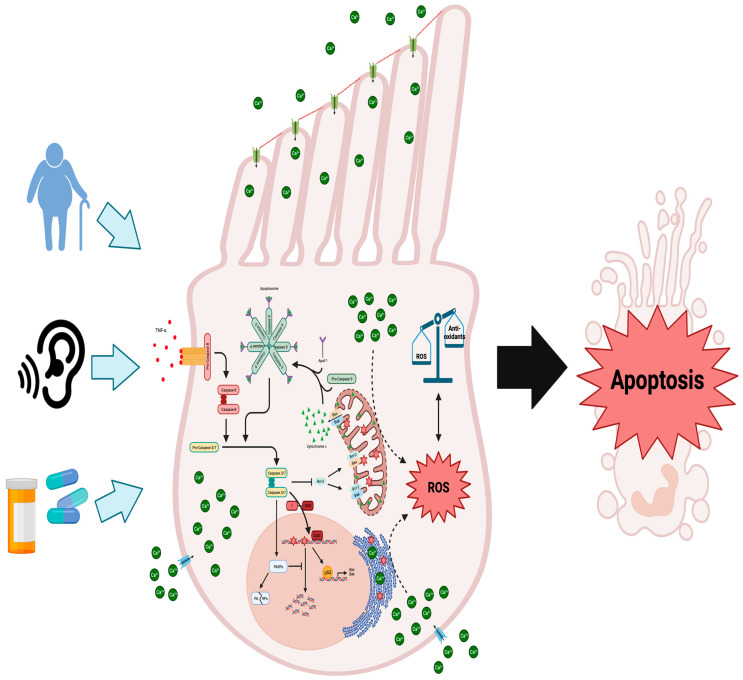
Molecular mechanisms underlying cochlear hair cell demise due to aging, noise exposure, or ototoxic medications: When cochlear hair cells are injured, the intrinsic and extrinsic apoptotic pathways are initiated, alongside the generation of ROS and disruptions to calcium balance. Damage to these cells triggers external inflammatory molecules like TNF-α to bind to their receptors on the cell membrane, initiating the cleavage and activation of procaspase 8. This sets off the extrinsic apoptotic cascade, activating effector caspases 3 and 7. Caspase 3 further triggers the degradation of anti-apoptotic Bcl-2 protein, releasing cytochrome c from mitochondria. It also activates CAD, leading to DNA fragmentation by cleaving its inhibitor ICAD. Typically, DNA repair is facilitated by PARPs, but caspases 3/7 inhibit this process, contributing to cellular degradation. Excessive DNA damage boosts the pro-apoptotic role of p53, upregulating genes like Bax and Bak. Furthermore, p53 directly interacts with Bak at the outer mitochondrial membrane, promoting cytochrome c release. Through the recruitment of procaspase 9 and Apaf-1, cytosolic cytochrome c assembles the apoptosome, which further activates caspases 3 and 7. Excessive ROS production surpassing antioxidant systems induces oxidative stress and DNA damage. Figure created with BioRender.com.

Prolonged or high dB noise exposure overwhelms this adaptive mechanism and induces permanent threshold shifts by the upregulation of pro-apoptotic Bcl-2 family member, Bak, that lead to hair cell loss [41]. Likewise, noise exposure upregulates endogenous proinflammatory cytokine and chemokine production through activation of nuclear factor kappa B (NF-kB) signaling and recruits immune cells to the cochlea [42,43,44]. Tumor necrosis factor alpha (TNF-α), interleukin 6 (IL-6), and interleukin 1-beta (IL-1β) expressed in response to excessive noise can overcome anti-apoptotic signals to induce hair cell death (Figure 1) [45,46]. In addition to apoptosis, necroptosis has been implicated in outer hair cell death through an upregulation of receptor-interacting serine/threonine protein kinases in chinchilla and mice cochleae [47,48]. Necroptosis varies from apoptosis by permeabilizing plasma and intracellular membranes releasing cellular components into the surrounding environment [48]. Upon release, the cellular components act as damage associated molecular patterns which can be recognized by Toll-like receptors and receptors for advanced glycation products potentiating the pro-apoptotic signaling [49,50]. Moreover, hair cell death is also regulated through the induction of intrinsic, pro-apoptotic caspase cascades.

The activation of intrinsic caspase cascades is initiated by the permeabilization of the mitochondria membrane and release of cytochrome c, which is crucial for the subsequent activation of downstream effector caspases 3 and 7 (Figure 1) [51]. This process involves the formation of the apoptosome, which consists of cytochrome c, Apaf-1, and procaspase 9 (Figure 1). The apoptosome then cleaves and activates the zymogens, procaspases 3 and 7, thereby initiating the apoptotic program [51]. Caspases 3 and 7 act as proteases that target essential cell survival proteins like Bcl-2 [52]. Bcl-2 plays a crucial role in preventing the oligomerization of the pro-apoptotic Bcl-2 family member, Bax, on the outer mitochondrial membrane (Figure 1). Bax recruits the smaller pro-apoptotic Bcl-2 family member, Bak, to the mitochondrial membrane, where both oligomerizes in conjunction [53].The oligomerization of both Bax and Bak forms pores that permeabilize the outer mitochondrial membrane, resulting in the release of cytochrome c, which is necessary for the activation of the intrinsic caspase cascade leading to apoptosis (Figure 1) [53,54,55]. Therefore, this mechanism of action creates a positive feedback loop, as the activation of the intrinsic caspase cascade leads to the degradation of Bcl-2, facilitating Bax-dependent cytochrome c release and further reinforcing the initial apoptotic signal.

In an auditory hair cell line, cisplatin treatment enhanced activation of both the intrinsic and extrinsic pathways as judged by the activation of caspase 8 and 9 (Figure 1) [56]. Further evidence for the role of the extrinsic and intrinsic pathways in hair cell loss comes from a study that exposed chinchillas to loud noise and found caspase 8 is activated alongside caspase 9 in hair cells in response to noise [57]. However, in cisplatin-treated guinea pigs, only caspase 9 was shown to be activated in cochlear hair cells [58]. Despite these results that show the extrinsic caspase cascade to be involved in NIHL, caspase 3 activation, downstream of both pathways, is evident and essential for caspase-dependent apoptosis in cochlear hair cells (Figure 1). Both in vitro and in vivo models display caspase 3 activation in response to cisplatin [58,59], aminoglycosides [60,61], and noise exposure [57,62]. Furthermore, selective inhibition of caspase 3 protects cochlear hair cells from cisplatin-induced apoptosis in vivo [58]. Supporting the role of the intrinsic pathway, in response to the same cochlear insults, Bax activation increases in cochlear hair cells of gerbils and guinea pigs treated with cisplatin and aged gerbils [58,63,64]. Cytochrome c release into the cytosol connects Bax activation and the intrinsic caspase cascade, which has been shown to occur in vivo in response to cisplatin [58], aminoglycoside [61], or noise exposure [57] (Figure 1). Concomitant with the release of cytochrome c, Bax oligomerization also alters the mitochondrial transmembrane potential and causes an increase in cytosolic reactive oxygen species (ROS) [49,55]. Increased generation of ROS overwhelms the antioxidant systems of the inner ear and offers another potential mechanism for cochlear hair cell death.

Accumulation of cytosolic ROS induces oxidative stress when endogenous antioxidant systems are downregulated or overwhelmed shifting the cell towards apoptosis. Aging, ototoxic drugs, and noise are all demonstrated to cause cochlear hair cell death and hearing loss through oxidative stress [65]. Additionally, genetic polymorphisms in antioxidant systems, namely, glutathione S-transferase [66], N-acetyltransferase 2 [67,68], and superoxide dismutase (SOD) 2 [69], predispose individuals to develop ARHL. In the CBA/J experimental model of ARHL, ROS increased alongside the decrease in SOD2 at 18 months of age [70]. This suggests that SNHL may be driven by oxidative stress-related DNA and mitochondrial damage. For instance, in the SAMP8 mouse model of ARHL, 8-oxoguanine, a marker of both nuclear and mitochondrial oxidized DNA, was detected in hair, supporting, and spiral ganglion cells of the cochlea [71]. In a follow-up study from the same laboratory, hydrogen peroxide was shown to induce DNA damage leading to increased auditory brainstem response (ABR) threshold shifts as compared to control mice of the same age [72]. Administration of EUK-207, a SOD/catalase mimetic, prevented threshold shift in SAMP8 mice, suggesting that exogenous antioxidants provide an effective avenue for SNHL prevention. In vitro, HEI-OC1 cells, a heterogenous cell line isolated from the mouse organ of Corti, exposed to hydrogen peroxide became senescent through alterations to the morphology and functionality of the mitochondria [73]. While oxidative stress-induced models of ARHL proceed through promoting senescence, NIHL may be caused through calcium-dependent oxidative stress-induced apoptosis (Figure 1). Exposure to noise increasing ROS in the inner ear was established in guinea pigs and corroborated after in C57BL/6J mice, which suggests that NIHL can be mediated through oxidative stress [74,75].

As indicated above, OHCs appear to be affected first, as opposed to IHCs, in the early progression of hearing loss, and this susceptibility may be attributed to lower antioxidant content [76]. Also, it is plausible that ROS are elevated in response to increased levels of cytosolic calcium increases, leading to OHC death, as has been shown in neurons [77]. Cytosolic calcium increase in neurons was shown to cause a loss of the mitochondrial transmembrane potential and undergo caspase-dependent apoptosis [78,79]. Similarly, in guinea pigs, it was found that noise-induced temporary threshold shifts led to an increase in cytosolic calcium levels in OHCs [80], leading to a rise in ROS production, potentially creating a positive feedback loop that accelerates the progression of NIHL (Figure 1). In the cochlea of guinea pigs, noise exposure was also shown to result in the generation of isoprostanes, vasoactive lipid peroxidation products, which cause ROS accumulation and induce ischemia in the inner ear, restricting blood flow to hair cells and further promoting ROS generation [81].

Unmitigated oxidative stress can have detrimental effects on the cellular environment, leading to damage in cellular membranes and DNA. Notably, it has been observed that 67% of patients with mitochondrial DNA (mtDNA) disorders will develop sensorineural hearing loss, supporting the idea that the accumulation of oxidative stress-induced mtDNA damage may contribute to this condition [82]. Given that oxidative stress is a fundamental factor in multiple pathologies, including type 2 diabetes mellitus (T2DM), emerging evidence supports a strong association between T2DM and SNHL. For instance, mothers that develop T2DM are at risk for mtDNA mutations that are then maternally inherited by their children. The presence of either a 10.4kb mtDNA deletion or point mutation in the mitochondrial tRNA (Leu) passed on from the mother caused all children to develop both T2DM and sensorineural deafness [83,84]. In the third generation born to mothers with mtDNA mutations, eight of the thirteen children developed sensorineural deafness and only two had both conditions [84]. These lineages suggest that oxidative stress in T2DM patients may be related to the development of SNHL.

## 3. Anti-Hearing Loss Therapies

Numerous therapeutic strategies have been identified to tackle hearing loss at the cellular and molecular levels. While research in pharmacological treatments for hearing loss is still in its infancy, several drugs have shown promise in clinical trials or preclinical studies targeting SNHL as well as various other hearing loss models (Table 2).

### 3.1. D-Methionine

As an antioxidant, D-methionine has been acknowledged for its potential therapeutic value in the prevention of certain types of hearing loss. For instance, in a phase II exploratory clinical trial, oral administration of D-methionine was examined for its safety and efficacy against cisplatin-induced ototoxicity in cancer patients. Patients receiving D-methionine prior to cisplatin treatment did not show significant threshold shifts at various testing frequencies in comparison to placebo. All documented adverse effects were found to be already common to cisplatin therapy. These results indicate that supplementation of D-methionine protects cancer patients against chemotherapeutic-induced hearing loss without added adverse effects [88], although the molecular mechanisms are yet to be fully elucidated. Pre-clinical investigations have substantiated the protective properties of D-methionine against NIHL. In chinchillas, select windows of D-methionine preloading effectively reduced ABR threshold shifts and increased certain antioxidant levels in both steady state and impulse noise exposures, thus revealing the otoprotective properties of D-methionine against NIHL [98]. These protective effects were corroborated in a randomized controlled trial, where D-methionine preloading demonstrated efficacy against NIH [89]. Similarly, post-administration of D-methionine was also able to protect against noise insults to the cochlea. Using a mouse model of NIHL, Wang et al. demonstrated that both pre- and post-administration of D-methionine treatment reduced cochlear hair cell loss, suppressed apoptosis, and preserved the expression of gap junction proteins, such as connexin 26 and 30 [99]. Additionally, several studies have highlighted the ability of D-methionine to modulate the activities of antioxidant enzymes, including SOD and catalase, thereby attenuating noise-induced oxidative stress [100,101]. However, it is important to note that while oxidative stress is a prominent factor in the pathology of hearing loss, recent ex vivo research has cautioned against high-dose antioxidant treatment. Cochlear explants subjected to prolonged exposure to high concentrations of antioxidants, such as D-methionine at 50 μM, exhibited adverse effects including severe damage to hair cell structure and complete loss of spiral ganglion neurons and supporting fibers [102].

### 3.2. Ebselen/SPI-1005

Ebselen (SPI-1005) stands out as a pharmaceutical candidate for treating hearing loss due to its unique mimicry of the antioxidant enzyme, glutathione peroxidase (GPx) 1 [103]. During phase II clinical testing in healthy adults, 400 mg of ebselen given orally twice daily was found effective and safe to reduce acute noise-induced hearing loss [90]. Other completed, yet unpublished, clinical trials have focused on investigating ebselen’s potential for treating Meniere’s disease, a rare disorder of the inner ear that can cause a range of hearing-related disturbances [104]. Phase Ib and IIb trials conducted in Meniere’s or Meniere’s disease-probable patients found that subjects treated with SPI-1005, in comparison to placebo, showed clinically relevant improvements of several symptoms that characterize Meniere’s disease [91]. Moreover, some preclinical data confer that ebselen may be beneficial for patients diagnosed with cystic fibrosis [105]. Tobramycin, an aminoglycoside, is commonly used long-term to help treat cystic fibrosis-associated pulmonary bacterial infections [106]. Unfortunately, tobramycin is known to induce ototoxicity [107,108]. Gu and colleagues, however, demonstrated that mice co-treated with ebselen are able to alleviate tobramycin-induced ototoxicity of the cochlea and co-treated cochlear explants were observed to have reduced tobramycin-induced hair cell loss [105]. Likewise, in a model of NIHL, ebselen protected rats from OHC loss and damage to the stria vascularis [103]. Kil and colleagues provided evidence suggesting that ebselen directly scavenges ROS/RNS and possibly upregulates or preserves endogenous GPx1. This hypothesis is supported by previous research demonstrating that ebselen preserves GPx1 levels in spiral ganglion cells exposed to peroxynitrite [109]. Although the precise molecular mechanisms remain unclear, it is plausible that ebselen’s protective effects against hearing loss primarily result from its ability to mitigate oxidative stress. Notably, ebselen has been shown to alleviate inflammation in db/db mice by inhibiting SHIP2 [110] and to attenuate renal damage through the upregulation of autophagy and sequential initiation of the nuclear factor erythroid 2-related factor-2 (Nrf2) pathway [111]. However, further research is needed to confirm whether these mechanisms contribute to its protective effects in hearing loss models.

### 3.3. Glucocorticoids

In 1980, a double-blind clinical trial revealed the efficacy of oral steroids in treating idiopathic sudden sensorineural hearing loss (ISSNHL) [112], and since, a wealth of clinical trials has investigated the most commonly prescribed steroid medicine for hearing loss, dexamethasone [113]. Various studies have indicated that dexamethasone protects against chemotherapeutic-induced hearing loss [85], mild-to-severe ISSNHL [86], and Meniere’s disease [87,114], among others. However, collective evidence on dexamethasone and its genuine impact on hearing disorders, or as preventative care, appears to be less conclusive. For instance, the benefits of intratympanic dexamethasone remain a controversial subject. Some evidence suggests intratympanic (IT) injection of dexamethasone is a resourceful route of treatment, as IT dexamethasone was reported as being easy to administer, even possibly safer than, and just as effective as IV administration [115,116,117]. Supportively, Ni and colleagues reported no significant differences in hearing improvements between routes of corticoid administration—specifically systemic, IT, or combination therapy—in patients with sudden deafness [118]. In contrast, according to others, IT administration of dexamethasone may be the riskier therapy. In another clinical trial of sudden hearing loss, two patients from the IT cohort reported having tears to the tympanic membrane [119]. Additionally, the true efficacy of dexamethasone has been under review, as Salt and Plontke brought forward that the pharmacokinetic properties of dexamethasone-phosphate, the pro-drug commonly used in clinical practice, may not be compatible with local drug delivery and thus would fail to be clinically relevant [120]. Other discrepancies such as higher incidence of postoperative retching [121] and null outcomes [122] have also been reported with dexamethasone therapy.

While direct human evidence remains scarce, preclinical investigations suggest that aldosterone might have the potential to decelerate or even halt the advancement of presbycusis. Halonen et al. conducted experiments on middle-aged CBA/CaJ mice to elucidate the otoprotective properties of this mineralocorticoid hormone. Their findings indicated that animals receiving long-term aldosterone treatment exhibited stabilized ABR thresholds. In contrast, the untreated group experienced a notable shift in noise threshold, increasing from 39 dB to 49 dB [123]. Further in vivo models have demonstrated that aldosterone [124], synthetic aldosterone, or a combination of aldosterone and prednisone at low doses [125,126] are also effective at restoring autoimmune mediated hearing loss.

### 3.4. Sodium Thiosulfate

Sodium thiosulfate, an FDA-approved drug, has emerged as a potential intervention to reduce the likelihood and severity of cisplatin-induced hearing loss in pediatric patients. Despite common side effects such as vomiting and nausea, sodium thiosulfate maintains an overall safety profile, making it a pertinent consideration for improving the quality of life in pediatric cancer patients undergoing cisplatin-based chemotherapy. Such perspective is supported by results from few animal studies [127,128] and human clinical trials. For example, in a study by Brock et al. [96], sodium thiosulfate administered six hours post-cisplatin chemotherapy in children with standard-risk hepatoblastoma demonstrated a lower incidence of hearing loss without compromising overall or event-free survival. Similarly, Rischin et al. highlighted the potential of DB-020 to reduce cisplatin-induced ototoxicity [97]. DB-020 is an intratympanic formulation of sodium thiosulfate [129]. Additionally, the role of sodium thiosulfate in preventing cisplatin-induced hearing loss is supported by a multicenter, randomized, phase 3 trial (ACCL0431), which found a significant decrease in the incidence of hearing loss among participants treated with sodium thiosulfate compared to the control group [95]. These findings, along with results from a prospective analysis comparing hearing loss incidence in a phase III randomized trial versus IV high-dose cisplatin chemoradiation without sodium thiosulfate, provide evidence supporting the potential of sodium thiosulfate to mitigate cisplatin-induced hearing loss in pediatric patients. The collective results emphasize the promising role of sodium thiosulfate in addressing this side effect and its potential impact on the well-being of pediatric cancer patients [94]. It is worth noting that while the molecular mechanisms mediating sodium thiosulfate autoprotective effects are yet to be determined, studies by Hochman and colleagues were unable to demonstrate that sodium thiosulfate can attenuate gentamicin-induced ototoxicity in mice [130]. This underscores the need for additional evidence and a more comprehensive understanding of sodium thiosulfate’s modes of action.

### 3.5. Additional Therapies

A phase 1 study tested the safety and audiometric effects of FX-322 for the treatment of SNHL. The authors reported that patients with chronic SNHL treated with an intratympanic injection of FX-322 exhibited clinically meaningful improvements in speech recognition and hearing function without drug intolerances [92]. Notably, clinical phase 2a trials are up and coming.

Moreover, IGF-1 has recently shown some early therapeutic promise for treating SNHL, yet symptoms such as pain, dizziness, and headache are all adverse effects reported by treated patients [93].

Tinnitus or phantom auditory perception is a condition that is frequently reported among patients with hearing loss [131,132]. A noncompetitive NMDA receptor antagonist, AM-101, was specifically evaluated for its efficacy and safety to treat several hearing loss pathologies. Some clinical outcomes have shown that intratympanic injections of AM-101 are well tolerated and safe with some patient-reported improvements in tinnitus-related symptoms [133,134,135]. However, in a placebo-controlled phase II trial, the primary end point to assess the efficacy of AM-101 for acute inner ear tinnitus was unsuccessful. Moreover, the benefit of AM-101 treatment failed to transfer to ISSNHL-related tinnitus patients, a subcategory of the total study population [133].

Because SNHL results from permanent damage to and loss of cochlear hair cells, a fair portion of therapeutic research has set out to explore the potential of hair cell regeneration [136]. Presently, three companies have advanced preclinical data into clinical works, and these have been outlined and well described in a recent review by Hinton et al. Briefly, clinical hair cell regeneration therapies primarily embrace gene therapy and small molecule inhibitor or combination approaches [137].

## 4. Phytochemicals as Medicine

Phytochemicals have begun to emerge as efficacious treatment options and adjuvants in Western medicine since biomedical research seeks alternatives to pharmaceuticals. Phytochemicals—non-nutrient bioactive compounds originating from natural sources, most commonly plants, but also including some fungi and algae compounds [138]—have been shown to be beneficial in combatting a plethora of chronic and non-chronic diseases across the board including cancer, aging, diabetes, osteoporosis, and cardiovascular disease. Phytochemicals exhibit potent antioxidative, anti-inflammatory, and neuroprotective characteristics attributed to their structural diversity and abundance [139]. Phytochemicals encompass various classes, including polyphenols, triterpenoids, steroids, saponins, and alkaloids, each containing distinct subgroups [138]. Among these, polyphenols are particularly noteworthy, as they are the most abundant antioxidants found in foods. They can be further categorized into flavonoids, phenolic acids, stilbenes, tannins, and coumarins. The clinical community has shown support for phytochemicals due to their demonstrated effectiveness with fewer side effects and lower costs compared to pharmaceuticals [139]. In the subsequent section, we will elucidate the protective properties of several phytochemicals against disruptions in cochlear hair cell homeostasis and functionality, along with their potential in mitigating hearing loss.

### 4.1. Phytochemicals in Sensorineural Hearing Loss

**Figure 2:**
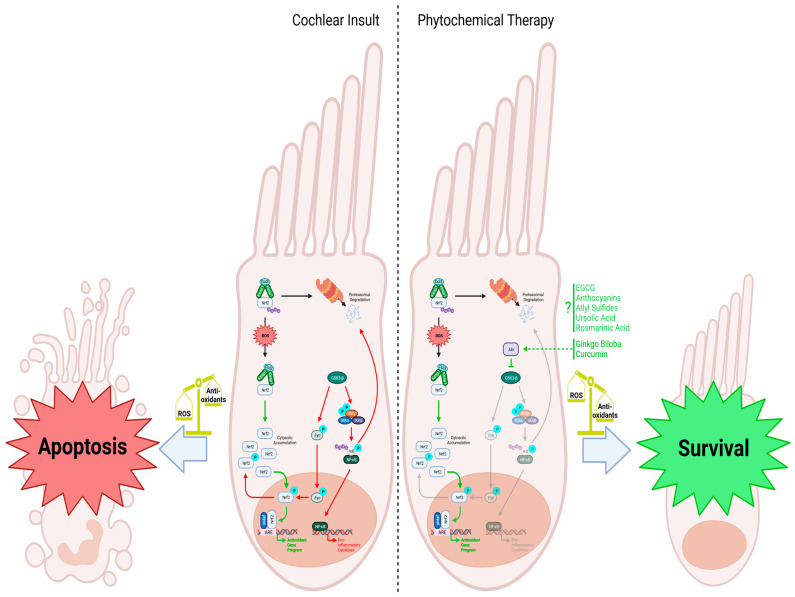
Cochlear insult: When cellular reactive oxygen species (ROS) are low, Nrf2 is degraded via proteasomal degradation facilitated by the Keap1 homodimer, which recruits the ubiquitin ligase Cul3 for subsequent ubiquitination. However, when cochlear hair cell injury occurs and ROS levels rise, Keap1 undergoes oxidation, leading to the accumulation of Nrf2 within the cytosol. This prompts Nrf2 to translocate into the nucleus, where it forms a complex with the leucine zipper-type transcription factor sMaf and binds to the antioxidant response element (ARE). This activation leads to the upregulation of antioxidant genes, counteracting the increased ROS levels in the cell. GSK3-β negatively regulates the Nrf2-dependent gene expression mechanism by phosphorylating Fyn, leading to its translocation into the nucleus, where it phosphorylates Nrf2. Phosphorylated Nrf2 is then exported from the nucleus back into the cytosol, inhibiting the antioxidant response. Additionally, GSK3-β activates the IKK complex and leads to the phosphorylation of IκB and its subsequent ubiquitination and degradation. The release of NF-κB and translocation to the nucleus promotes the transcription of proinflammatory cytokines that further worsens cell injury. The reduced antioxidant response, combined with the upregulation of proinflammatory cytokines, makes the cell more susceptible to apoptosis. Phytochemical therapy involving substances like rosmarinic acid, *Ginkgo biloba*, and curcumin enhances the cellular response to increased ROS levels. *Ginkgo biloba* and curcumin activate Akt, inhibiting the GSK3-β-induced phosphorylation cascade. Without Fyn stimulating Nrf2 nuclear exclusion, Nrf2 can activate the antioxidant program, helping to scavenge ROS. Although various phytochemicals are known to stimulate Nrf2 and the transcription of its downstream antioxidant genes, specific mechanisms are still being investigated. Nevertheless, administering these phytochemicals effectively combats oxidative stress, promoting the survival and function of cochlear hair cells. Figure created with BioRender.com.

The activation of antioxidant and anti-inflammatory pathways, such as Nrf2 and inhibition of NF-κB, is crucial for mitigating the harmful effects that lead to cochlear hair cell death (Figure 2). Additionally, maintaining proper blood flow to the cochlea is essential for nutrient circulation and waste removal, as any disruption in this process can result in damage to the hair and supporting cells. Phytochemicals have been found to influence these pathways, offering potential benefits for chronic diseases that share similar etiology with SNHL, such as cardiovascular disease, nephropathy, and T2DM. In this section, we will explore the body of research on specific phytochemicals and their effects on SNHL, including those that have demonstrated efficacy in treating T2DM. Given the association between T2DM and SNHL, it is possible that phytochemicals could be used to address both conditions and potentially improve outcomes for individuals with these health concerns.

#### 4.1.1. *Ginkgo biloba*

*Ginkgo biloba* stands out as one of the most thoroughly investigated extracts for the prevention and treatment of hearing loss, yet its clinical effectiveness continues to be a subject of debate. Multiple clinical trials and several animal models have shown beneficial results for its use in ARHL, NIHL, and hearing loss induced by cisplatin. In human studies, supplementation with *Ginkgo biloba* extract EGb 761 during chemotherapy with cisplatin showed significant hearing protection, as measured by distortion product otoacoustic emission (DPOAE) tests, compared to the control group [140]. Similarly, in another trial using Ginkocer, a *Ginkgo biloba* supplement, ARHL patients experienced an average of 15.56% improvement in hearing [141]. Moreover, coal miners exposed to blast noise damage and treated with *Ginkgo biloba* through a tympanic injection, in combination with hyperbaric oxygen therapy, exhibited significant improvement in acoustic response rates compared to those who only received traditional therapy [142].

These clinical findings are supported by in vivo evidence from various animal models. For instance, *Ginkgo biloba* supplementation to cisplatin-exposed rats showed significantly improved DPOAE tests compared to the cisplatin group and resulted in fewer apoptotic cells in the organ of Corti and stria vascularis [143]. These results corroborate previous investigations employing rats and guinea pigs as experimental models of cisplatin-induced ototoxicity [144,145], further substantiating the effectiveness of *Ginkgo biloba* in ameliorating cochlear dysfunction induced by cisplatin. Notably, *Ginkgo biloba’s* antioxidant properties also protected guinea pigs from ototoxicity caused by pesticides and antibiotic-induced ototoxicity [146,147]. In models of NIHL, the prophylactic use of EGb 761 in Mongolian gerbils reduced NIHL and prevented permanent threshold shift [148,149]. Additionally, in C57BL/6 mice, a combination of cilostazol and EGb 761, called Renexin^®^, protected the organ of Corti from degeneration and NIHL, suggesting potential for reversing permanent NIHL [150]. Furthermore, in studies investigating ARHL in young and aged rats, EGb 761 correlated with reduced caspase 3/7 activity and threshold shifts [151].

Mechanistically, *Ginkgo biloba* exhibits a diverse range of actions that contribute to its effectiveness in various models of SNHL. In vitro studies exploring its effects on neural stem cells (NSCs) demonstrated *Ginkgo biloba*’s ability to promote NSC proliferation and differentiation into functional neurons [152]. When exposed to oxidative stress induced by hydrogen peroxide, *Ginkgo biloba*’s antioxidant effects appeared to protect NSCs by preventing mitochondrial depolarization and apoptosis through modulation of the intrinsic apoptotic pathway, as evidenced by the upregulation of Bcl-2 and downregulation of Bax and Caspase-3 [153]. Similarly, in HEI-OC1 cells treated with cisplatin, Ginkgolide B exhibited comparable results by inhibiting the intrinsic pathway of apoptosis. These effects were attributed to reduced ROS levels via downregulation of NADPH oxidase 2 expression and upregulation of Akt/Nrf2/HO-1 signaling (Figure 2) [154]. Moreover, EGb 761 was proposed to operate through the upregulation of sirtuin 1 (SIRT1), which was found to alleviate SNHL induced by 3-nitropropionic acid in rats, potentially protecting fibrocytes and spiral ganglion cells through SIRT1 expression [155]. Additionally, EGb 761 preserved the organ of Corti from gap junction intercellular communication disturbance by inhibiting cisplatin-induced downregulation of connexins [156]. The observed anti-inflammatory effects of *Ginkgo biloba* were evident in a rat model of NIHL, where it inhibited IL-1β, IL-6, TNF-α, and cyclooxygenase (COX) 2 while increasing heat shock protein 70 and heat shock factor 1, ultimately reducing NIHL [157]. EGb 761 was also found to lower total oxidant status and DNA damage in NIHL while reducing threshold shifts. Consistent with these findings, when Renexin^®^, a pharmaceutical formulation comprising cilostazol and *Ginkgo biloba* leaf extract, known for its decongestant and appetite suppressant properties, as well as its potential in alleviating ischemic symptoms associated with peripheral arterial disease [158], was administrated to a rat model of cisplatin-induced hearing loss, a significant decline in ROS levels and apoptosis was observed [159]. Nevertheless, it is imperative to underscore that there exists conflicting evidence regarding the efficacy of *Ginkgo biloba.* Indeed, investigations involving guinea pigs and zebrafish have documented either a lack of therapeutic response or even adverse effects when *Ginkgo biloba* is administered concurrently with the antibiotic amikacin [160,161,162]. Furthermore, a clinical trial focusing on the treatment of ARHL using *Ginkgo biloba* and other phytochemicals failed to demonstrate statistically significant alterations in hearing thresholds [163]. These contradictory outcomes suggest that the efficacy of *Ginkgo biloba* in managing SNHL may be contingent upon the specific experimental model and context in which it is employed. Further research is warranted to elucidate the determinants influencing its efficacy and to delineate the optimal conditions for its therapeutic application.

#### 4.1.2. Resveratrol

Resveratrol, a naturally occurring polyphenol classified as a stilbenoid and found in over 70 plant species [164], has been subject to extensive research aiming at unveiling its therapeutic potential against SNHL. Various mechanisms have been proposed to elucidate its efficacy across diverse experimental models. For example, in a rat model of cisplatin-induced hearing impairment, the co-administration of resveratrol with cisplatin resulted in a significant reduction in ABR thresholds concomitant with improvement in DPOAEs compared to cisplatin treatment alone [165,166]. Resveratrol has also shown protective effects on the inner ear, reducing damage and lowering ROS levels in rats and guinea pigs, respectively [167,168]. Furthermore, in a model of diabetes-induced ototoxicity in rats, resveratrol at multiple dosages decreased apoptosis in the inner ear, as evidenced by reduced caspase-3, Bax, and TUNEL staining [169]. Notably, resveratrol was effective against amikacin-induced ototoxicity, in contrast to *Ginkgo biloba* [170]. In other studies, resveratrol’s protection against cisplatin-induced hearing loss was suggested to involve modulation of the PTEN/PI3K/Akt pathway, resulting in the upregulation of miRNA-455-5p [171]. Furthermore, resveratrol decreased proinflammatory gene expression after cisplatin treatment [172]. However, at high doses, resveratrol may potentiate cisplatin-induced ototoxicity [173]. It is worth noting that the protective effects of resveratrol are not limited to its antioxidant properties, as it has been also shown to regulate gene expression to ameliorate the effects of ototoxic drugs. For example, in aminoglycoside-induced ototoxicity, resveratrol upregulates antioxidant enzymes to relieve oxidative stress while downregulating proinflammatory genes [174]. Additionally, resveratrol has been identified as a potent SIRT1 agonist, and its induction of SIRT1/PGC1-α signaling pathways has been shown to ameliorate the effects of oxidative stress in guinea pig models of NIHL and C57BL/6 mice exposed to noise [175,176]. Furthermore, resveratrol has been proposed to protect against NIHL by inhibiting serotonin transporters to increase serotonin levels [177]. The attenuation of COX2 expression may also play a role in reducing threshold shifts after noise exposure [178].

In models of NIHL, resveratrol has been shown to lower ABR threshold shifts and protect the cochlea from acoustic damage [179,180]. Resveratrol has been observed to decrease the expression of pro-apoptotic genes, such as Bax and Bak, while concurrently upregulating anti-apoptotic genes, including Bcl-2 and Bcl-xL [181]. Furthermore, in the same study, the treatment with resveratrol resulted in the downregulation of inflammatory and apoptotic genes, such as NF-κB, COX2; inducible nitric oxide synthase (iNOS); as well as caspases 3 and 9. These effects seem to be mediated by SIRT1, an NAD+-dependent deacetylase that was shown to exhibit a protective role in aging cochlea [182]. Notably, resveratrol protected mice from hair cell loss through the induction of the miRNA-34-a/SIRT1/p53 signaling pathway [183]. These observations were substantiated in a recent investigation, where resveratrol was demonstrated to elicit the same signaling cascade to preserve aging cochlear hair cells, spiral ganglion cells, and the stria vascularis [184]. Furthermore, resveratrol modulates the involvement of autophagy in aging by regulating SIRT1 expression [185].

#### 4.1.3. Curcumin

Due to curcumin’s limited bioavailability, researchers have explored nanoencapsulation as a targeted delivery method to the cochlea. Studies in guinea pigs have demonstrated that nanoparticle-encapsulated curcumin can be more effective than dexamethasone in preventing hearing loss, indicating its potential as a promising therapeutic option [186]. Nanoparticles have also shown safety and efficacy in mitigating cochlear damage induced by cisplatin [187]. Furthermore, curcumin has proven effective in a model of gentamicin-induced ototoxicity and has demonstrated potential as a prophylactic agent, with similar protective effects observed in the gentamicin-induced ototoxicity model [188]. In rat models of ototoxicity induced by the chemotherapeutic agent paclitaxel or cisplatin, curcumin administered prior to or in combination with the drugs alleviated cochlear damage [189,190]. Rat models of NIHL have also shown that curcumin induces Nrf2 signaling, offering protection against DPOAE shifts and loss of inner and outer hair cells (Figure 2) [189,191].

Although the specific molecular mechanisms of curcumin’s action are not fully elucidated, several pathways have been identified. Following noise exposure, curcumin treatment protected the cochlea by reducing NF-κB activation [192]. Curcumin has also been found to protect supporting tissue in the cochlea by decreasing the expression of calcineurin, NFATc1, and overall lowering the apoptotic index [193]. Another possible mechanism involves the inhibition of IKKβ phosphorylation that suppresses CXCL5 and the induction of MKP-1, which negatively regulates p38-MAPK signaling [194]. Interestingly, curcumin has been shown to preserve cisplatin’s action against tumors while protecting against ototoxicity by activating STAT3 and Nrf2 in vivo (Figure 2) [195]. Additionally, curcumin may regulate HO-1 expression and block the JNK-mediated cell death pathway to preserve cochlear cells [196,197]. Overall, while the precise mechanisms of curcumin’s action require further investigation, the accumulating evidence suggests its potential as a therapeutic agent for various forms of hearing loss.

#### 4.1.4. *Salvia miltiorrhiza* (Danshen)

*Salvia miltiorrhiza*, also known as Danshen in China, is commonly used therapeutically in an injectable form. It has shown promise in protecting against cisplatin-induced ototoxicity in guinea pigs by reducing damage to the cochlea and spiral ganglion through the attenuation of iNOS expression [198]. *Salvia miltiorrhiza* has also been found to safeguard against ABR threshold shifts when kanamycin is used to induce ototoxicity, without compromising the antibiotic’s efficacy [199]. The effectiveness of *Salvia miltiorrhiza* in combatting hearing loss appears to be linked to its regulation of iNOS expression.

For instance, in a guinea pig model of aminoglycoside-induced hearing loss, *Salvia miltiorrhiza* was found to attenuate the expression of iNOS and caspase 3, resulting in a decrease in the number of apoptotic cells [200]. Similarly, *Salvia miltiorrhiza* was shown to exhibit protective effects against gentamicin-induced damage to the stria vascularis through reducing iNOS activity [201]. Additionally, it seems that *Salvia miltiorrhiza*’s ability to reduce acetylcholinesterase may contribute to protecting the cochlea and spiral ganglion cells after streptomycin administration [202]. Corroborating these findings, another study demonstrated a reduction in iNOS activity in response to streptomycin when cotreated with *Salvia miltiorrhiza* [203]. An interesting alternative mechanism of *Salvia miltiorrhiza*’s action was observed in guinea pigs, where it increased superoxide dismutase activity and reduced lipid peroxidation, leading to improvements in auditory function [204].

#### 4.1.5. Epigallocatechin Gallate (EGCG)

EGCG has been touted as an effective treatment to a wide variety of conditions. As with curcumin, EGCG has been packaged into nanoparticles, providing enhanced protection against damage to the cochlea in C57/BL6 mice [205]. It is worth noting, however, that EGCG demonstrated protection of spiral ganglion cells appears to deviate from the mechanisms employed by other phytochemicals. Unlike many, EGCG does not predominantly rely on the upregulation of antioxidant systems, as evidenced by its ability to suppress SOD2 expression [206]. Instead, EGCG may exert its effectiveness through the regulation of the STAT1/STAT3 axis. Studies using mice and rats with cisplatin-induced ototoxicity revealed that EGCG’s protective effects involve the suppression of STAT1 activity while preserving the function of STAT3 and Bcl-xL to reduce outer hair cell death [207,208,209]. EGCG also appears to act through other pathways to reduce oxidative stress and inflammation. Investigations in aminoglycoside ototoxicity using the network pharmacology approach have shown that EGCG induces a cellular response involving Akt1, dihydrofolate reductase, and the hepatocyte growth factor receptor product of the proto-oncogene MET, which helps preserve outer hair cells [210]. Moreover, EGCG has been demonstrated to prevent hair cell loss in the organ of Corti by inhibiting NF-κB and caspase-3 activity in a study of NO ototoxicity [211]. Additionally, EGCG has shown promise in promoting hearing recovery by increasing neural stem cell proliferation and differentiation [212]. Notably, it has also been identified as a novel inhibitor of Notch signaling, which may contribute to hair cell proliferation [213]. In summary, EGCG exhibits a multifaceted approach in protecting against hearing loss, involving the regulation of various molecular pathways and cellular responses, making it a promising candidate for potential therapeutic applications in hearing-related conditions.

#### 4.1.6. *Panax ginseng*

*Panax ginseng* exerts protection against medication, palmitate, ischemia, and noise-induced cochlear hair cell damage through a class of saponins referred to as ginsenosides. Largely, *Panax ginseng* is studied as a whole-food supplement and research into isolated ginsenosides has offered some insight into the molecular mechanisms of action. Sprague–Dawley rats exposed to gentamicin had increased hearing thresholds and significant stereocilia damage [214]. When rats were supplemented with Korean red ginseng (KRG) for one week prior and during exposure to gentamicin, researchers observed a significant improvement in hearing thresholds and reduction in cochlear hair cell damage [214]. In rats exposed to prolonged high-decibel noise, supplementation with KRG protected against cell death in both the organ of Corti and spiral ganglion [215]. In an effort to understand the effects of isolated ginsenosides, Fujita and colleagues demonstrated ginsenoside-Rb1 reduces the loss of spiral ganglion cells after the induction of cochlear ischemia by occlusion [216].

In vitro studies conducted in HEI-OC1 and UB/OC-2 cochlear cell lines better characterized the molecular effects of the cochlear insults and the protection offered by *P. ginseng* and its bioactive components. Exposure to cisplatin shifted HEI-OC1 cells to undergo apoptosis due to the generation of ROS and proinflammatory protein expression. Solubilized KRG prevented these pro-apoptotic processes by attenuating ROS generation and the activation of NF-κB inflammatory signaling [217,218]. Kim and co-authors proposed that the mitigation of inflammation occurs through the attenuation of phosphorylation and activation of IKK, leading to a reduction in the transcriptional activity of NF-κB [218]. Additionally, cochlear hair cell death can be induced through the activation of the unfolded protein response and ER stress. Palmitate induces apoptosis in UB/OC-2 cells through the activation of ER stress and inflammation in addition to increasing the generation of ROS [219]. The inhibition of only one of these stress pathways is insufficient to block cell death; however, ginsenoside-Rc protected against palmitate-induced cell death through the amelioration of all three pathways [219]. An alternative mechanism of protection also includes a reduction of PARP cleavage after gentamicin exposure in HEI-OC1 cells treated with ginsenoside-Rb1 and -Rb2 [214]. While clinical human data validating the potential role of ginsenosides in mitigating hearing loss are limited, the existing evidence aligns with the concept that *Panax ginseng* provides protection against cochlear damage. Textile workers are exposed to continuous, high-decibel occupational noise, which makes them an interesting population to study the efficacy of *P. ginseng*. In a study spanning 14 days, textile workers were administered oral doses of ginseng, with hearing thresholds assessed both prior to and following the trial [220]. The results indicated that ginseng provided defense against noise-induced temporary threshold shifts, although its efficacy was surpassed by supplementation with N-acetyl-cysteine [220]. Additional long-term studies are needed to ascertain whether *Panax ginseng* confers protection against permanent threshold shifts, which arise following cochlear hair cell and spiral ganglion cell damage induced by factors such as noise exposure, medication, aging, or diabetes.

#### 4.1.7. Astragalosides

Astragalosides have primarily been investigated in the context of NIHL, specifically in acute impulse studies. A clinical trial investigating the effectiveness of astragalosides in recovering from acute acoustic trauma revealed that the group supplemented with astragalosides experienced significantly shorter recovery times [221]. In comparison to other antioxidants, namely, α-lipoic acid and vitamin E, astragalosides and α-lipoic acid were notably more effective in reducing ABR thresholds and protecting hair cells, outperforming vitamin E [222]. In guinea pigs subjected to impulse noise exposure, administration of astragalosides led to a significant reduction in ABR thresholds, hair cell damage, and the expression of iNOS and reactive nitrogen species (RNS) production [223]. These observations are corroborated by another investigation, which indicated that astragalosides protect cochlear hair cells by mitigating ROS levels and suppressing caspase-3 activity [224]. Furthermore, astragalosides were demonstrated to maintain the integrity of connexin 26 and KCNQ1, thereby safeguarding the stria vascularis in guinea pigs [225]. Additionally, astragalosides effectively mitigated cisplatin-induced ototoxicity by preserving outer hair cells through the reduction of malondialdehyde and nitric oxide levels [226].

#### 4.1.8. Garlic

Several primary bioactive components present in garlic, including S-allylmercaptocysteine (SAMC), diallyl disulfide (DD), and S-allylcysteine, have demonstrated effectiveness in treating SNHL, as well as other forms of HL [227,228,229,230]. In studies involving rats with gentamicin-induced ototoxicity, all three components effectively mitigated the condition, as evidenced by reductions in ABR thresholds [228]. However, SAMC and DD exhibited greater potency than S-allylcysteine. Additionally, rats supplemented with garlic in their diet were protected from gentamicin-induced BERA threshold shifts [229]. Moreover, in a rat model of noise-induced hearing loss, SAMC and DD were found to be more effective than vitamin B12 in protecting the cochlea from noise-induced damage [230]. Likewise, the use of MEKRITEN, a garlic extract liquid formulated by the Tashkent Pharmaceutical Institute and promoted for its therapeutic benefits, has shown efficacy in healing suppurative otitis with fewer side effects compared to commonly used treatments in patients [227]. Although the exact mechanism of action for garlic and its derivatives is not fully understood, the current proposed mechanism seems to involve the attenuation of caspase-dependent and PARP-1/AIF-dependent cell death [231]. For instance, allicin, a garlic product, was observed to reduce caspase-3 cleavage, PARP-1, and AIF nuclear translocation in the stria vascularis [231]. Similarly, in mice with cisplatin-induced ototoxicity, allicin protected spiral ganglion neurons by reducing cytochrome c release and the expression of Bax, caspases 3 and 9, and p53 [232]. Allicin also upregulated SOD, thereby preserving the outer hair and supporting cells of the cochlea [232].

#### 4.1.9. Additional Phytochemicals

**Figure 3:**
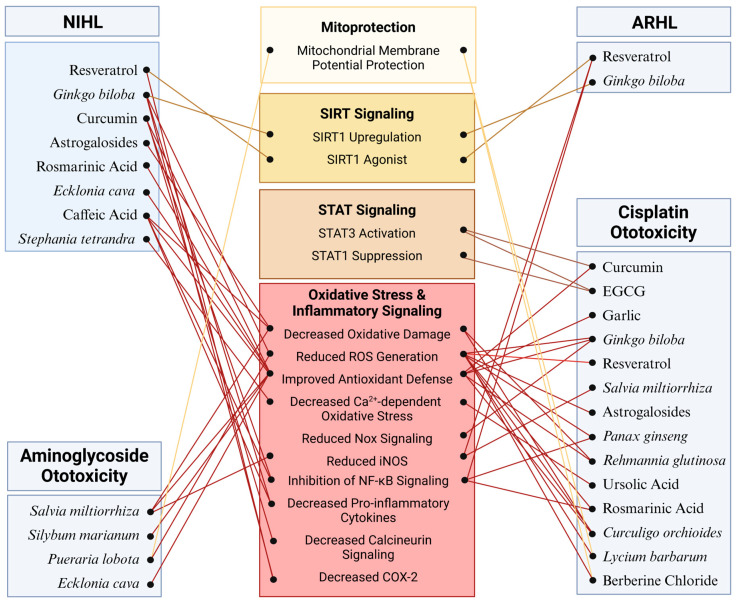
Molecular mechanisms mediating the potential protective effects of phytochemicals against SNHL: The effectiveness of phytochemicals has been evaluated across various models of SNHL with particular emphasis on cisplatin ototoxicity. Across these models, phytochemicals demonstrate a protective effect against cochlear damage primarily by regulating oxidative stress and inflammatory pathways. Additionally, phytochemicals such as resveratrol and curcumin provide protection by activating SIRT and STAT signaling pathways, respectively. Further research is warranted to elucidate additional specific targets mediating the effects of phytochemicals in the cochlea. Figure created with BioRender.com.

Many additional phytochemicals (Table 3) have been investigated for their potential uses as primary strategies to preserve auditory function or combat the ototoxicity of medications while maintaining drug efficacy. Largely, these phytochemicals act through similar mechanisms to the ones above by attenuating ROS generation either by upregulation of endogenous antioxidant enzymes in cochlear hair cells or by sequestration of free radicals by the phytochemical itself (Figure 3). Many have anti-inflammatory effects as well, thereby reducing NF-κB activation and expression of interleukin cytokine species (Figure 3) [233,234]. Ameliorating oxidative stress and inflammatory signaling combats apoptotic signaling that can be directly observed through the inhibition of caspase 3 and PARP cleavage as well as mitochondrial apoptotic signaling [233,235,236]. Novel mechanisms of action for hearing preservation have also been elucidated in these investigations. Ursolic acid was shown to reduce oxidative stress through the inhibition of the TRPV1/Ca^2+^/calpain pathway that is activated during cisplatin-induced ototoxicity [237]. Silymarin, the bioactive component in milk thistle (*Silybum marianum*), protected against gentamicin damage by restoring NGF production and upregulating its receptor, Trk-A [238]. Puerarin, isolated from *Pueraria lobota*, reduced threshold shift after noise exposure in mice through its action to decrease PKCγ expression and increase GABAB receptor 1 and 2 expression [239]. Berberine chloride acts on spiral ganglion cells to reduce cytomegalovirus apoptosis by reducing Nox3-dependent ROS generation (Figure 3) [240]. The wealth of phytochemical species and mechanisms of action constitute an arsenal for the preservation of hearing across all classes of SNHL.

## 5. Conclusions

In summary, hearing loss poses a complex and widespread challenge affecting 20% of the global population. In this review, we delineated the extent, clinical variations, and current understanding of the molecular foundations of SNHL. We elucidated how the molecular pathophysiology of SNHL, particularly involving the induction of oxidative stress and inflammation, parallels that of other pathological conditions. Within this context, phytochemicals have emerged as promising candidates for addressing hearing loss, leveraging their antioxidant and anti-inflammatory properties, potentially offering a safer avenue for mitigating hearing loss compared to strategies involving hair cell regeneration or gene therapy. Furthermore, we provided an up-to-date exploration of the diverse molecular pathways influenced by these compounds. Because phytochemicals do modulate more than one molecular pathway, and can also act epigenetically, comprehensive studies of their effects in different biochemical contexts should be combined with clinical investigations to elucidate their potential in mitigating hearing loss. As discussed above, a wealth of preliminary studies suggests that investigation of phytochemicals for hearing loss treatment holds significant promise. Notably, further exploration, and efforts to enhance their bioavailability, delivery, efficacy, and safety, should also be pursued.

## Figures and Tables

**Table 1:** Global Burden of Disease Expert Group in Hearing Loss recommended classification of hearing loss types.

Classification	Pure-Tone Audiometry (PTA)	Treatment
Normal hearing	−10.0–19.9 decibels (dB)	None
Mild hearing loss	20.0–34.9 dB	OTC hearing aids and assisted listening devices
Moderate hearing loss	35.0–49.9 dB	Hearing aids
Moderately severe hearing loss	50.0–64.9 dB	Hearing aids
Severe hearing loss	65.0–79.9 dB	Hearing aids; cochlear implants
Profound hearing loss	80.0–94.9 dB	Cochlear implants
Complete or total hearing loss	≥95.0 dB	Cochlear implants
Unilateral hearing loss	<20.0 dB in better ear with ≥35.0 dB in worse ear	OTC hearing aids and assisted listening devices

**Table 2:** Exploring hearing loss therapies: clinical trials and key findings.

Treatment	Type of Trial	Model of Hearing Loss	Treatment/Regimen	Major Conclusion(s)	Reference(s)
**Dexamethasone**	Prospective randomized controlled trial (NCT01372904)	Cisplatin-induced hearing loss	Unilateral intratympanic (IT) dexamethasone (0.7–1.0 mL of 10 mg/mL) injection prior to cisplatin (3–11 sessions)	IT dexamethasone treatment attenuated cisplatin-induced hearing dysfunction	[85]
	Prospective, quasi-randomized, multicenter clinical trial (NCT NR)	Idiopathic sudden sensorineural hearing loss	Intravenous (IV) prednisolone (75 mg/day for 3 days then tapered down over 12 days) or prednisolone with 3 IT dexamethasone (0.4 to 0.6 mL of 4 mg/mL) injections over 5 days	Combination therapy was more effective to treat ISSNHL than IV prednisolone alone	[86]
	Prospective clinical trial (NCT NR)	Meniere’s disease-related hearing loss	Unilateral IT dexamethasone (4 mg/mL) injections (5 injections)	IT injections prevents the progression of Meniere’s disease-related hearing loss	[87]
**D-methionine**	Randomized parallel double-blind placebo-controlled exploratory phase 2 study (NCT NR)	Cisplatin-induced hearing loss	100 mg/kg oral supplementation of D-methionine or placebo 1 h before cisplatin administration	Oral supplementation protected against cisplatin-induced hearing loss	[88]
	Randomized, placebo-controlled clinical trial	Noise-induced hearing loss	Oral D-methionine tablet pre-loading	Oral D-methionine prior to noise exposure protected against prolonged auditory threshold shift	[89]
**Ebselen (SPI-1005)**	Randomized, double-blind, placebo-controlled phase 2 trial (NCT01444846)	Noise-induced hearing loss	Oral dose, twice daily of 200 mg, 400 mg, or 600 mg ebselen twice a day for 4 days, starting 2 days before the sound challenge	400 mg of ebselen (2×/day) is safe and preventative against NIHL	[90]
	Phase 1b multi-center RCT (NCT02603081)	Meniere’s disease-related HL	Oral dose of ebselen, 3 capsules (200 mg, 400 mg, or 600 mg) twice daily before am and pm meals for 21 days	Ebselen-treated patients had a positive response to treatment in some MD-related symptoms	Reviewed in [91]
	Phase 2b multi-center RCT (NCT03325790)	Meniere’s disease-related HL	Oral dose of 200/400 mg ebselen twice daily for 28 days	Ebselen-treated patients had clinically relevant improvements across various audiometric effects	Reviewed in [91]
**FX-322**	Phase 1b prospective randomized, double-blind, placebo controlled clinical trial (NCT03616223)	Sensorineural hearing loss	Unilateral, single-dose of IT FX-322 (0.05 mL or 0.2 mL) or placebo (0.05 mL or 0.2 mL)	FX-322 had clinically meaningful audiometric effects in patients with SNHL	[92]
**Insulin-like growth factor-1 (IGF-1)**	Randomized controlled trial (NCT NR)	Sensorineural hearing loss	IT administration (0.5 mL) of IGF-1 or IGF-1-soaked Gelfoam	IT and Gelfoam IGF-1 improved hearing thresholds in patients with SNHL	[93]
**Sodium thiosulfate**	Randomized, prospective phase 3 trial (NCT NR)	Cisplatin with chemoradiation-induced hearing loss	IV sodium thiosulfate (9 g/m^2^/30 min then 12 g/m^2^/2 h) concurrent with intra-arterial cisplatin on days 1, 8, 15, 22 as well as radiation therapy	IV sodium thiosulfate offers protection against speech frequency hearing loss due to cisplatin administration and chemoradiation	[94]
	Open-label, randomized phase 3 trial (NCT00716976)	Cisplatin-induced hearing loss	IV sodium thiosulfate (16 g/m^2^) 6 h after cisplatin administration	IV sodium thiosulfate administration reduces the incidence of cisplatin-induced hearing loss	[95]
	Multi-center open-label randomized phase 3 trail (NCT00652132, EudraCT 2007-002402-21)	Cisplatin-induced hearing loss	IV sodium thiosulfate (20 g/m^2^/15 min) 6 h after cisplatin administration (4 preoperative and 2 post-operative)	Lower incidence of cisplatin-induced hearing loss was observed in patients receiving sodium thiosulfate	[96]
	Phase 1b randomized, double-blind, placebo-controlled, multicenter study (NCT04262336)	Cisplatin-induced hearing loss	IT DB-020 (12% or 25%) administered in one ear and placebo in other (randomized/blinded) 3 h before cisplatin treatment	IT administration of DB-020 reduces cisplatin ototoxicity compared to placebo	[97]

**Table 3:** Exploring the effects of select phytochemicals in the prevention and treatment of hearing loss.

Phytochemical Origin	Constituent Tested	Constituent Classification	Model of Hearing Loss	Hearing Assessment	Reported Outcome(s)	Reported Mechanism(s)	Reference(s)
***Drynaria fortunei***	Flavonoid fraction	Flavonoid	Gentamicin ototoxicity in guinea pigs	Auditory brainstem response	Lower hearing threshold shifts and significantly less damage to inner and outer hair cells	Not determined	[241]
***Rehmannia glutinosa***	Steamed root ethanol extract	N/A	Cisplatin ototoxicity in HEI-OC1 cells	N/A	Reduced HEI-OC1 cell apoptosis in a dose-dependent manner	Decreased lipid peroxidation and improved scavenging activity against free radicals	[242]
**Purified compound**	Ursolic acid	Triterpenoid	Cisplatin ototoxicity in mice	Auditory brainstem response	Reduced hearing threshold shifts and outer hair cell damage	Inhibits the TRPV1/Ca^2+^/calpain oxidative stress pathway	[237]
	Ursolic acid	Triterpenoid	Hydrogen peroxide ototoxicity in HEI-OC1 cells	N/A	Attenuated HEI-OC1 cell damage and apoptosis	Reduced lipid peroxidation and induced catalase and glutathione peroxidase activity	[243]
**Purified compound**	Rosmarinic Acid	Caffeic acid ester	Cisplatin ototoxicity in mice, rat cochlear explants, and HEI-OC1 cells	Auditory brainstem response	Reduced threshold shift and protected cochlear hair cells and HEI-OC1 cells from cisplatin-induced apoptosis.	Inhibited caspase 1 and its downstream targets caspase 3 and 9 as well as cytochrome c release and translocation of AIF. Bax expression was downregulated and Bcl-2 was upregulated along with reductions in ROS generation and NF-kB activation.	[233]
	Rosmarinic acid	Caffeic acid ester	NIHL in rats	Auditory brainstem response	Preserved cochlear hair cells and reduced noise-induced threshold shift	Potentiated Nrf2/HO-1 signaling, which upregulates superoxide dismutase for antioxidant defense	[244]
	Rosmarinic acid	Caffeic acid ester	NIHL in rats	Auditory brainstem response	Significant decrease of oxidative stress in the cochlea and reduced NIHL	N/A	[245]
***Curculigo orchioides***	Rhizome ethanol extract	N/A	NIHL in mice	Auditory brainstem response	Reduced hearing threshold shifts, auditory system damage, and cochlear function deficits	N/A	[246]
	Root ethanol extract	N/A	Cisplatin ototoxicity in mice and HEI-OC1 cells	Auditory brainstem response	Attenuated cochlear and peripheral auditory function impairments and HEI-OC1 apoptosis in a dose-dependent manner	Reduced ROS generation and lipid peroxidation through scavenging activity against free radicals	[247]
***Lycium barbarum***	Wolfberry extract	Polysaccharide	Cisplatin ototoxicity in rat organ of Corti explants	N/A	Attenuated cochlear hair cell apoptosis	Reduced ROS generation and change in the mitochondrial membrane potential	[248]
***Silybum marianum***	Silymarin	Flavonoid	D-galactose-induced ARHL in rats	Auditory brainstem response	Reduced threshold shift	N/A	[249]
	Silymarin	Flavonoid	NIHL in guinea pigs	Auditory brainstem response	Reduced temporary and permanent threshold shifts	N/A	[250]
	Silymarin	Flavonoid	Gentamicin ototoxicity in guinea pigs	Auditory brainstem response	Attenuated threshold shift and cochlear hair cell apoptosis	Reduced oxidative stress by increasing catalase activity and restored production of NGF and expression of tropomyosin-related kinase receptor-A	[238]
	Silymarin	Flavonoid	Cisplatin ototoxicity in HEI-OC1 cells	N/A	Reduced HEI-OC1 cell apoptosis	Inhibited caspase 3 and PARP cleavage and attenuated cell cycle arrest	[235]
***Pueraria lobota***	Puerarin	Isoflavone	NIHL in mice	Auditory brainstem response and distortion product otoacoustic emission	Reduced ABR threshold shift but no effect on DPOAE signal	Decreased expression of PKCγ and increased expression of GABAB receptor 1 and 2 in the cochlear nuclei complex	[239]
	Puerarin	Isoflavone	Gentamicin ototoxicity in mice and HEI-OC1 cells	Auditory brainstem response	Reduced hearing threshold, cochlear hair cell damage, and HEI-OC1 apoptosis	Attenuated ROS production and inhibited mitochondrial-dependent apoptotic signaling	[236]
***Ecklonia cava***	Phlorofucofuroeckol A and dieckol	Phlorotannin	NIHL in mice	Auditory brainstem response	Reduced ABR threshold shift after noise exposure and increased cochlear hair cell survival	N/A	[251]
	Polyphenolic extract	Polyphenol	NIHL in mice	Auditory brainstem response	Reduced temporary threshold shift	Induction of free radical scavenging activity	[252]
	Dieckol	Phlorotannin	Gentamicin ototoxicity in mouse cochlear explants	N/A	Reduced cochlear hair cell loss	Induction of free radical scavenging activity	[253]
**Purified compound**	Caffeic acid phenethyl ester	Phenol	Gentamicin ototoxicity in rats	Auditory brainstem response and distortion product otoacoustic emission	Reduces ABR threshold shift, DPOAE signals deterioration, and cochlear degeneration	N/A	[254]
	Caffeic acid	Hydroxycinnamic acid	NIHL in rats	Auditory brainstem responses	Reduces ABR threshold shift and cochlear hair cell death	Decreased NF-κB, IL-1β, and oxidative/nitrosative damage through the upregulation of Nrf2/HO-1	[234]
	Caffeic acid phenethyl ester	Phenol	Cisplatin ototoxicity in rats	Distortion product otoacoustic emission	Reduced DPOAE signal deterioration and outer hair cell death	N/A	[255]
	Caffeic acid phenethyl ester	Phenol	Streptomycin ototoxicity in rats	Distortion product otoacoustic emission	Reduced DPOAE signal deterioration and cochlear hair cell death	N/A	[256]
	Caffeic acid phenethyl ester	Phenol	Cisplatin ototoxicity in rats	Distortion product otoacoustic emission	Reduced DPOAE signal deterioration	Reduced plasma xanthine oxidase activity	[257]
***Stephania tetrandra***	Tetrandrine	Bis-benzylisoquinoline alkaloid	NIHL in mice	Auditory brainstem response and distortion product otoacoustic emission	Reduced ABR threshold shifts, DPOAE signal deterioration, outer hair cell damage, and inner hair cell/spiral ganglion synapse loss	Amelioration of transient Ca^2+^ current in spiral ganglion cells in a dose-dependent manner	[258]
***Crocus sativus***	Endophytic exopolysaccharide	Polysaccharide	Gentamicin ototoxicity in zebrafish lateral line and HEI-OC1 cells	N/A	Increased hair cell survival in zebrafish and reduced HEI-OC1 cell damage	N/A	[259]
**Purified compound**	Berberine chloride	Benzylisoquinoline alkaloid	NIHL in guinea pigs	Auditory brainstem response	Outer hair cell-targeted nanoparticle therapy increases berberine delivery to outer hair cells and reduces threshold shifts and preserved outer hair cell integrity	N/A	[260]
	Berberine chloride	Benzylisoquinoline alkaloid	Cisplatin ototoxicity in mouse cochlear explants	N/A	Reduced hair cell damage	Reduced ROS generation, DNA fragmentation, and preserved mitochondrial membrane potential	[261]
	Berberine chloride	Benzylisoquinoline alkaloid	Cytomegalovirus-induced apoptosis in spiral ganglion cells	N/A	Reduced apoptosis in spiral ganglion cells	Reduced N-methyl-D-aspartate-type receptor/Nox3-induced mitochondrial ROS generation	[240]

## Data Availability

Not applicable.
